# Serum Trimethylamine N-Oxide as a Diagnostic and Prognostic Biomarker in Dogs with Chronic Kidney Disease: A Pilot Study

**DOI:** 10.3390/ani15152170

**Published:** 2025-07-23

**Authors:** Seung-Ju Kang, Wan-Gyu Kim, Keon Kim, Chang-Hyeon Choi, Jong-Hwan Park, Seog-Jin Kang, Chang-Min Lee, Yoon Jung Do, Woong-Bin Ro

**Affiliations:** 1 College of Veterinary Medicine and BK21 FOUR Program, Chonnam National University, Gwangju 61186, Republic of Korea; darkfantasy0705@naver.com (S.-J.K.); namootneep@naver.com (W.-G.K.); gun8883@naver.com (K.K.); cch5013@gmail.com (C.-H.C.); jonpark94@naver.com (J.-H.P.); cmlee1122@jnu.ac.kr (C.-M.L.); 2 Animal Diseases & Health Division, National Institute of Animal Science, Rural Development Administration, Wanju-gun 55365, Republic of Korea; hijin@korea.kr

**Keywords:** chronic kidney disease, dogs, trimethyl-amine N-oxide

## Abstract

Trimethylamine N-oxide (TMAO) is known to increase in human cardiovascular, metabolic, and renal diseases. In human medicine, TMAO has recently been utilized as a diagnostic and prognostic biomarker for renal dysfunction, and research is ongoing regarding its potential as a therapeutic target. This study aimed to evaluate the diagnostic and prognostic potential of TMAO as a supportive biomarker in dogs with chronic kidney disease (CKD). To assess its diagnostic utility, TMAO concentrations were compared between a CKD group (*n* = 32) and a healthy control group (*n* = 32). In addition, patients with CKD were subdivided into stages 2 (*n* = 12), 3 (*n* = 11), and 4 (*n* = 9) and compared individually with the healthy controls. For prognostic evaluation, the CKD group was monitored over six months, and the TMAO levels were compared between survivors (*n* = 18) and non-survivors (*n* = 14). The TMAO concentrations showed a highly significant difference between patients with CKD and healthy controls (*p* < 0.0001). Patients with each different CKD stage exhibited statistically significant differences compared with the healthy controls (*p* < 0.05). Furthermore, the median TMAO levels tended to increase with advancing CKD stage; however, the differences among stages were not statistically significant. In addition, within the CKD group, TMAO concentrations were significantly higher in non-survivors than in survivors at the six-month follow-up (*p* = 0.0142). This pilot study highlights the potential of TMAO as a supportive renal biomarker for diagnostic and prognostic evaluation in canine CKD.

## Simple Summary

Trimethylamine N-oxide (TMAO) is a gut microbiota-derived metabolite that has been widely studied in human medicine due to its association with cardiovascular and renal diseases. Although it has shown promise as a biomarker for early detection and prognosis in human kidney dysfunction, its clinical utility in veterinary medicine has not yet been explored. This study investigated the potential of TMAO as a supportive biomarker in dogs with chronic kidney disease (CKD). Serum TMAO levels were significantly higher in dogs with CKD than in healthy controls, and the levels tended to increase with the progression of CKD stage. Furthermore, TMAO concentrations were significantly elevated in non-survivors compared to survivors during a six-month follow-up. These findings suggest that TMAO may serve as a useful biomarker for both the diagnosis and prognosis of canine CKD, offering a novel tool for the clinical evaluation and management of renal disease in veterinary practice.

## 1. Introduction

Chronic kidney disease (CKD) is defined as structural or functional abnormalities of one or both kidneys that persist for more than three months [1]. The etiology of CKD is diverse, including familial, congenital, and acquired factors [2]. One study reported a CKD prevalence of 33% in dogs aged eight years or older, indicating that CKD prevalence increases with age [3]. The diagnosis and staging of CKD follow the guidelines established by the International Renal Interest Society (IRIS) and are based primarily on serum creatinine and symmetric dimethylarginine (SDMA) concentrations.

The conventional renal biomarkers include creatinine, SDMA, and cystatin C. Creatinine is widely used for diagnosing CKD in humans and animals [1]. However, factors such as muscle mass, the consumption of high-protein diets, and vitamin C supplementation can influence creatinine levels, resulting in reported false-positive and false-negative outcomes [4]. SDMA has been increasingly utilized recently for CKD diagnosis in animals. Unlike creatinine, SDMA is less influenced by muscle mass. Nevertheless, some studies have indicated that SDMA concentrations may still be affected by conditions such as diabetes mellitus, sepsis, and thyroid disorders [5]. Cystatin C is frequently used as a biomarker in humans but is less commonly utilized in veterinary medicine. It is produced by all nucleated cells and serves as a sensitive indicator of the glomerular filtration rate (GFR) [6]. However, cystatin C levels may vary with age, breed, and body fat percentage [7]. Furthermore, non-renal conditions such as inflammation, thyroid disorders, and neoplasia can also elevate cystatin C concentrations [7].

Trimethylamine N-oxide (TMAO) is a metabolite produced through oxidation by hepatic flavin-containing monooxygenase 3 (FMO3) after dietary precursors such as choline and L-carnitine are converted into trimethylamine (TMA) by gut microbial TMA lyase [8]. TMAO is not metabolized in mammals and is predominantly excreted by the kidneys [9]. Elevated TMAO levels have been associated with cardiovascular diseases as well as diabetes mellitus, dyslipidemia, and renal disease. Furthermore, increased TMAO levels are correlated not only with a higher long-term mortality risk but also with elevated inflammatory markers, highlighting the potential utility of TMAO for early diagnosis, prognostic assessment, and as a therapeutic target [10,11,12].

This study aimed to evaluate the advantages of measuring TMAO to compensate for the limitations of the existing renal biomarkers. When used in conjunction with conventional renal biomarkers, TMAO compensates for the limitations of conventional renal biomarkers caused by muscle mass, protein intake, and other extrarenal factors. TMAO can serve as a significant prognostic indicator in patients with CKD. Its production by the gut microbiota also suggests its potential use in therapeutic targeting and monitoring through dietary modulation or probiotic intervention [13,14]. TMAO is detectable in the blood with high sensitivity and specificity, thus allowing for the potential monitoring of treatment efficacy and recovery. For these reasons, TMAO is increasingly measured alongside creatinine and cystatin C in patients with renal failure in human medicine. In this study, for the first time, we evaluated the diagnostic utility of serum TMAO in canine CKD. We additionally analyzed the correlation between TMAO and other renal biomarkers, investigated its association with various factors such as age, antibiotic usage, gastrointestinal (GI) symptoms, and dietary influences, and examined the role of serum TMAO as a biomarker for prognostic monitoring in canine CKD.

## 2. Materials and Methods

### 2.1. Serum Sampling

Serum samples for TMAO measurement were obtained from dogs diagnosed with CKD that visited our veterinary teaching hospital. All blood samples were collected at the time of diagnosis and centrifuged within 1 h at 12,000 RPM for 3 min. The serum surplus was stored at −20 °C until analysis. This experimental design was approved by the Institutional Animal Care and Use Committee of Chonnam National University (Approval No. CNU IACUC-YB-2025-33).

### 2.2. Animals

The CKD group (*n* = 32) was retrospectively selected from dogs diagnosed with CKD at our veterinary teaching hospital. The diagnosis and staging of CKD were performed according to the IRIS guidelines and were based on elevated serum creatinine or SDMA concentrations (Catalyst Dx Chemistry Analyzer, IDEXX Veterinary Diagnostics Co., Westbrook, ME, USA) without prerenal factors, persistently elevated SDMA levels above 14 µg/dL, abnormal renal imaging findings (Prosound a7, Hitachi Aloka Medical, Ltd., Tokyo, Japan), and persistent proteinuria. Disease staging was determined based on the higher value between creatinine and SDMA concentrations. All ultrasonographic examinations were performed by an experienced veterinary radiologist, and structural renal abnormalities observed on diagnostic imaging were considered indicative of CKD.

The control group (*n* = 30) included healthy dogs without any abnormalities based on medical history, physical examination, blood tests (complete blood count and serum biochemistry), and diagnostic imaging (radiography and abdominal ultrasound). Dogs with tumors, infections, acute kidney injury, cardiovascular diseases, or systemic inflammatory diseases, as well as animals under one year of age, were excluded.

### 2.3. Laboratory Examinations

The serum TMAO concentration was measured using a commercially available canine-specific ELISA kit (Catalog No. MBS7271760, MyBioSource, San Diego, CA, USA) based on a competitive enzyme immunoassay principle. All procedures were conducted according to the manufacturer’s instructions. Briefly, 100 μL serum samples pre-diluted 1:4 with sample diluent were added to antibody-precoated wells of a 96-well microtiter plate. Then, 50 μL of enzyme-conjugated TMAO-HRP solution was added. No balancing solution was used, as serum was the sample type. The plate was gently mixed and incubated at 37 °C for 1 h. Following incubation, the wells were washed five times with 1× wash buffer to remove unbound materials. Next, 50 μL each of Substrate A and Substrate B were added to all wells, and the plate was incubated in the dark at 37 °C for 15 min. The reaction was terminated by adding 50 μL of stop solution to each well. The optical density (OD) was immediately measured at 450 nm using a microplate reader (BioTek, Winooski, VT, USA). All samples were tested in duplicate, and the assay was performed twice on the same day to ensure intra-assay consistency. TMAO concentrations were calculated by interpolation from a standard curve generated using a four-parameter logistic (4-PL) regression model. The assay showed a strong correlation between OD values and recombinant canine TMAO protein standards (R^2^ = 0.9800, *p* < 0.001).

A serum chemistry analyzer (Catalyst Dx Chemistry Analyzer, IDEXX Veterinary Diagnostics Co., Westbrook, ME, USA) was used to quantify other serological enzymes such as creatinine, SDMA, blood urea nitrogen (BUN), alanine aminotransferase (ALT), alkaline phosphatase (ALP), gamma-glutamyl transferase (GGT) and total bilirubin (TBIL). The cystatin C, high-density lipoprotein (HDL), and low-density lipoprotein (LDL) assays were performed using an auto chemistry colorimetric analyzer (Dotto 2000 Auto Chemistry Analyzer, MTD Diagnostics Co., San Nicola la Strada, Italy) with cystatin C reagent (LC Diagnostics Co., Seoul, Republic of Korea).

### 2.4. Statistical Analysis

All statistical data were analyzed using commercially available software (GraphPad Prism v9.0, GraphPad Software Inc., San Diego, CA, USA). The Shapiro–Wilk test was performed to determine normality. For normally distributed data, parametric Student’s *t*-tests were performed to evaluate the statistical significance of various biomarkers between the CKD and control groups and to identify significant differences within the CKD group according to various factors. For non-normally distributed data, Mann–Whitney U tests were conducted. Pearson’s correlation analysis was used to evaluate the correlations between TMAO and conventional renal biomarkers. Furthermore, receiver operating characteristic (ROC) curves were used to compare the diagnostic sensitivity and specificity of TMAO with those of conventional renal biomarkers and to assess the predictive value of TMAO for 6-month prognosis. The statistical significance was set at *p* < 0.05 for all analyses.

## 3. Results

### 3.1. Study Population

Thirty-two dogs diagnosed with CKD were included in this study. The mean age was 11.12 ± 4.62 years. The most common breed was Pomeranian (*n* = 8), followed by Maltese (*n* = 4), Jindo (*n* = 4), Poodle (*n* = 3), Yorkshire Terrier (*n* = 2), Pekingese (*n* = 2), Golden Retriever (*n* = 2), Cocker Spaniel (*n* = 2), Bull Terrier (*n* = 1), Bichon Frise (*n* = 1), Coton de Tulear (*n* = 1), and two mixed-breed dogs. Of the CKD group (*n* = 32), 17 dogs were male (5 neutered), and 15 were female (2 spayed). When categorized according to CKD stage, dogs were classified as stage 2 (*n* = 12), stage 3 (*n* = 11), and stage 4 (*n* = 9).

The control group (*n* = 30) consisted of the following breeds: Beagle (*n* = 16), Maltese (*n* = 6), Bichon Frise (*n* = 2), Pomeranian (*n* = 2), Poodle (*n* = 1), Yorkshire Terrier (*n* = 1), Chihuahua (*n* = 1), and Schnauzer (*n* = 1); the mean age was 5.93 ± 3.89 years (Table 1).

### 3.2. Activity of Serum TMAO, Creatinine, SDMA and Cystatin C in Control Dogs and Dogs with CKD

**Figure 1:**
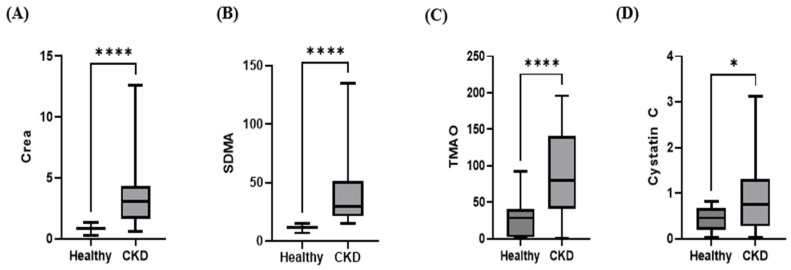
Box plot comparing the median creatinine (**A**), SDMA (**B**), TMAO (**C**), and cystatin C (**D**) concentrations between control dogs (*n* = 30) and dogs with CKD (*n* = 32). Serum TMAO, creatinine, and SDMA levels in the CKD group were significantly higher than those in the control group (Mann–Whitney U test; *p* < 0.0001) (median, 79.64 ng/mL; IQR, 41.12–140.8 versus median, 28.66 ng/mL; IQR, 1.708–40.52). The cystatin C levels in the CKD group were higher than those in the control group (Mann–Whitney U test; *p* < 0.05). * *p* < 0.05 and **** *p* < 0.0001.

The serum TMAO, creatinine, and SDMA concentrations were significantly higher in the CKD group than in the control group, and cystatin C was also significantly elevated in the CKD group compared with the control group. The serum creatinine concentration in CKD dogs was also significantly increased compared with the controls (CKD group: median 3.050 mg/dL, interquartile range (IQR): 1.625–4.350; control group: median 0.800 mg/dL, IQR: 0.7500–0.9500; *p* < 0.0001; Figure 1A). Similarly, the serum SDMA concentration was significantly higher in CKD dogs than in the controls (CKD group: median 29.50 µg/dL, IQR: 21.25–51.50; control group: median 12.00 µg/dL, IQR: 10.50–13.00; *p* < 0.0001; Figure 1B). The median serum TMAO concentration was significantly higher in CKD dogs than in the controls (CKD group: median 79.64 ng/mL, interquartile range [IQR]: 41.12–140.8; control group: median 28.66 ng/mL, IQR: 1.708–40.52; *p* < 0.0001; Figure 1C). Furthermore, cystatin C concentrations were significantly elevated in the CKD group compared with the control group (CKD group: median 0.7450 mg/L, IQR: 0.2750–1.313; control group: median 0.4550 mg/L, IQR: 0.1950–0.6675; *p* = 0.0135; Figure 1D).

### 3.3. Correlation of Serum TMAO with Creatinine, SDMA and Cystatin C Levels

**Figure 2:**
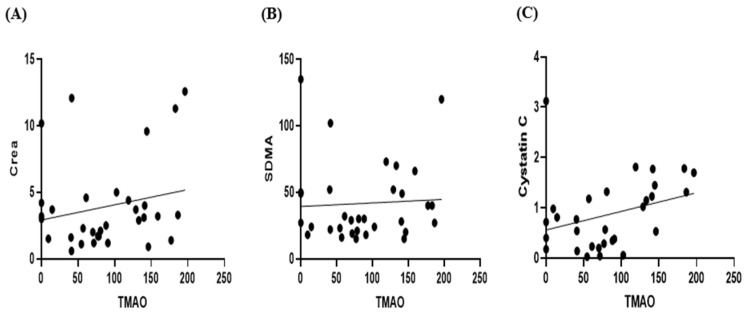
Scatter plots of serum TMAO concentration with creatinine (**A**), SDMA (**B**) and Cystatin C (**C**). Pearson’s correlation analysis displayed no correlation between serum TMAO and creatinine, SDMA and cystatin C (*p* = 0.2630, 0.7716, and 0.1006, respectively).

A correlation analysis of serum TMAO concentrations with serum creatinine, SDMA, and cystatin C showed no statistically significant relationships; however, there was a trend indicating that as TMAO concentrations increased, creatinine, SDMA, and cystatin C levels also tended to increase (Figure 2).

### 3.4. Evaluation of Sensitivity and Specificity

In this study, the optimal cut-off values obtained from the ROC curve analysis were 53.78 ng/mL for TMAO, 1.350 mg/dL for creatinine, 14.50 µg/dL for SDMA, and 0.7950 mg/L for cystatin C. At these cut-off points, the specificity was 86.67% for TMAO, 100% for creatinine, 96.55% for SDMA, and 80% for cystatin C, while the sensitivity was 71.88% for TMAO, 84.38% for creatinine, 100% for SDMA, and 36.67% for cystatin C (Table 2).

### 3.5. Comparison of Diagnostic Accuracy Through the ROC Curve Analysis

**Figure 3:**
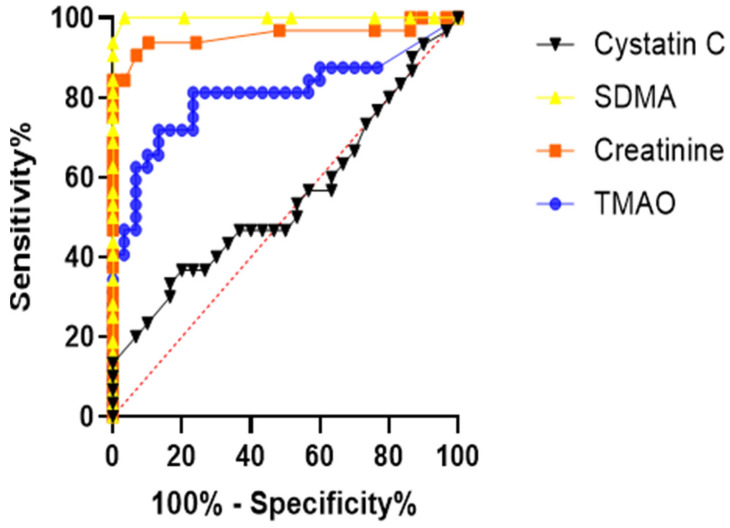
ROC curves of TMAO, creatinine, SDMA, and cystatin C concentrations to confirm their diagnostic accuracy in dogs with CKD. ROC curve for predicting CKD; TMAO (AUC = 0.8073), creatinine (AUC = 0.9558), SDMA (AUC = 0.9989) and cystatin C (AUC = 0.6844) compared with healthy dogs.

In the ROC curve analysis, the area under the curve (AUC) was confirmed to be 0.8073 for TMAO and 0.6844 for cystatin C. Reflecting their roles as serological gold standards for CKD, creatinine and SDMA exhibited AUC values of 0.9558 and 0.9989, respectively (Figure 3).

### 3.6. Comparison of the Median TMAO Concentration Among Control Dogs and Dogs with CKD Stage 2, 3, and 4

**Figure 4:**
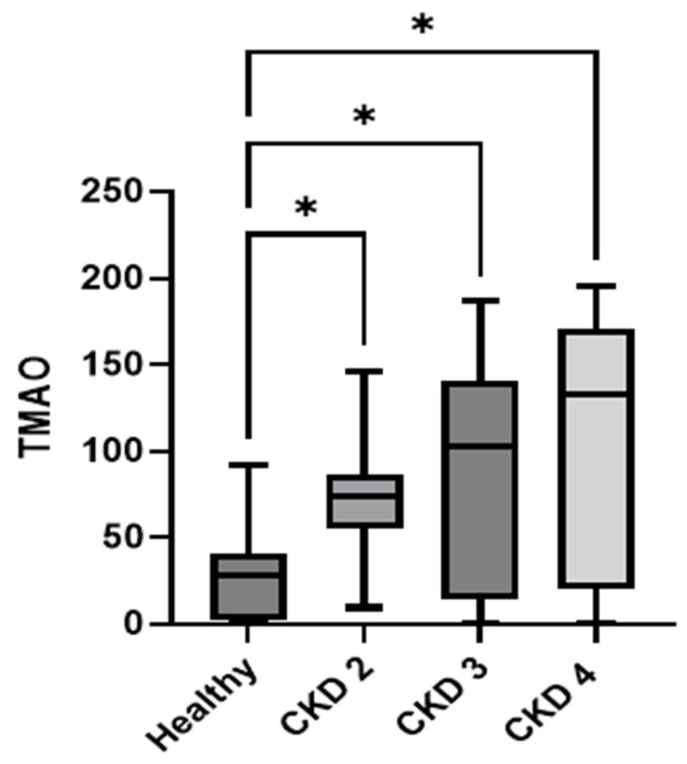
Box plot comparing the median TMAO concentration among control dogs (*n* = 30) and dogs with CKD stage 2 (*n* = 12), 3 (*n* = 11), and 4 (*n* = 9) dogs. TMAO levels were significantly higher in dogs with CKD stages 2, 3, and 4 than in healthy controls (*p* < 0.05 *). As presented in Table 1, the post hoc power analysis revealed that the minimum required sample size for adequate statistical power is 26 participants. Our current study utilized a total sample size of 62 participants, which substantially exceeds this requirement and ensures sufficient statistical power for the ANOVA analysis. This analysis demonstrates that the ANOVA results presented in Figure 4 are based on statistically adequate sample sizes with sufficient power to detect meaningful effects. We have incorporated these power analysis results into the manuscript to enhance the transparency and statistical rigor of our findings.

Among the 32 dogs diagnosed with CKD, the median serum TMAO concentrations in dogs with CKD stages 2, 3, and 4 were significantly higher than those in the controls (CKD stage 2: median 74.30 ng/mL, IQR: 55.07–86.40; CKD stage 3: median 102.6 ng/mL, IQR: 14.65–141.0; CKD stage 4: median 133.0 ng/mL, IQR: 20.63–171.0; control group: median 28.66 ng/mL, IQR: 1.708–40.52; *p* = 0.0005). The median TMAO values tended to increase with disease progression; however, these differences among CKD stages were not statistically significant (Figure 4).

### 3.7. Correlation and Comparison of Other Variables Between the Control Group and the CKD Group

**Figure 5:**
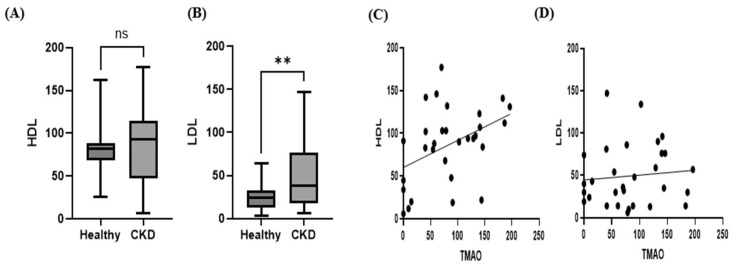
Box plot comparing the median HDL (**A**) and LDL concentrations (**B**) between control dogs (*n* = 30) and dogs with CKD (*n* = 30). Scatter plots of serum TMAO concentrations with HDL (**C**) and LDL concentrations (**D**). Serum LDL concentrations in the CKD group were significantly higher than those in the control group (Mann–Whitney U test; ** *p* < 0.01 in CKD) and serum HDL concentrations in the CKD group were not statistically significantly lower than those in the control group (Mann–Whitney U test; *p* > 0.05). Pearson’s correlation analysis for dogs with CKD showed that TMAO levels had a mild positive correlation with HDL (r = 0.4189, *p* = 0.0212) within the CKD group, and no correlation (*p* = 0.6228) was found between serum TMAO and LDL within the CKD group.

The correlation between TMAO and LDL was not statistically significant (Pearson correlation coefficient r = 0.09357, *p* = 0.6228). However, a weak positive correlation was observed between TMAO and HDL (Pearson correlation coefficient r = 0.4189, *p* = 0.0212). While HDL concentrations showed no significant difference between dogs with CKD and healthy controls, the LDL concentrations were significantly different between these two groups (*p* < 0.01) (Figure 5).

**Figure 6:**
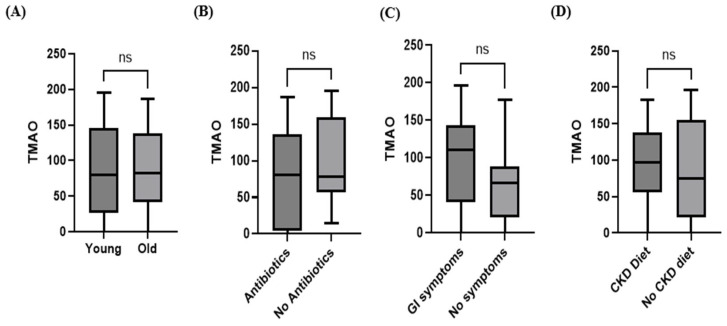
Box plot comparing the median concentrations of TMAO (**A**) between the young group (*n* = 12) and the old group (*n* = 20), (**B**) between the antibiotic-treated group (*n* = 17) and the untreated group (*n* = 15), (**C**) between the group with GI symptoms (*n* = 20) and the group without symptoms (*n* = 12), and (**D**) between the renal diet group (*n* = 16) and the normal diet group (*n* = 16). There was no statistically significant increase in TMAO concentrations based on age, antibiotic administration, the presence of GI symptoms, or renal diet status within the CKD group (Mann–Whitney U test; *p* > 0.05).

The median serum TMAO concentration in dogs with CKD showed no significant differences between older and younger dogs (older: median 79.64 ng/mL; younger: median 82.67 ng/mL; *p* = 0.6941; Figure 6A), between dogs not receiving antibiotics and those receiving antibiotics (without antibiotics: median 78.59 ng/mL; with antibiotics: median 80.69 ng/mL; *p* = 0.3074; Figure 6B), between dogs with GI symptoms and those without GI symptoms (with GI symptoms: median 110.8 ng/mL; without GI symptoms: median 66.29 ng/mL; *p* = 0.1915; Figure 6C), and between dogs fed a renal diet and those receiving a normal diet (renal diet: median 96.65 ng/mL; normal diet: median 74.30 ng/mL; *p* = 0.6894; Figure 6D).

**Figure 7:**
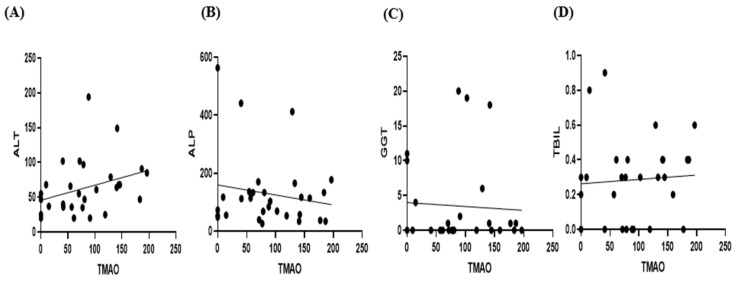
Scatter plots showing the TMAO concentration in relation to ALT ((**A**), *n* = 29), ALP ((**B**), *n* = 30), GGT ((**C**), *n* = 27), and TBIL levels ((**D**), *n* = 28) within the CKD group. Pearson’s correlation analysis revealed no significant correlations between TMAO and ALT, ALP, GGT, or TBIL within the CKD group.

The Pearson correlation coefficients between serum TMAO concentrations and the ALT, ALP, GGT, and TBIL levels were r = 0.2623 (*p* = 0.1615), r = −0.1740 (*p* = 0.3492), r = −0.0008521 (*p* = 0.9650), and r = −0.2348 (*p* = 0.2202), respectively, indicating no statistically significant correlations (Figure 7).

### 3.8. Prognostic Evaluation at Six Months Using Serum TMAO, Creatinine, SDMA, Cystatin C, HDL, and LDL Levels Within the CKD Group

**Figure 8:**
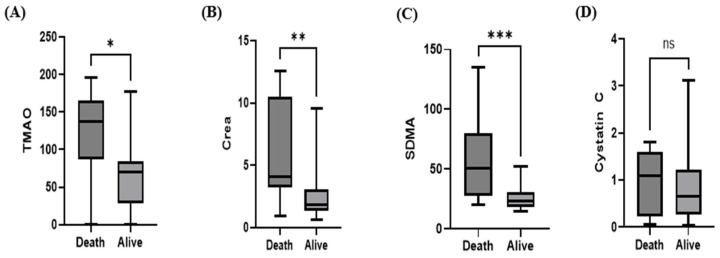
Box plot comparing the median TMAO (**A**), creatinine (**B**), SDMA (**C**) and cystatin C (**D**) concentrations between the non-survivor group (*n* = 14) and survivor group (*n* = 18) at a 6-month follow-up in the CKD group. Serum TMAO, creatinine, and SDMA levels in non-survivors were significantly higher than in survivors in the CKD group (Mann–Whitney U test; * *p* < 0.05; 0.0142, ** *p* < 0.01 and *** *p* < 0.001 in non-survivors), (TMAO median, 137.0 ng/mL; IQR, 87.26– 165.2 versus median, 70.18 ng/mL; IQR, 27.86–84.50). Serum cystatin C in non-survivors was not statistically significantly higher than in survivors within the CKD group (Mann–Whitney U test; *p* > 0.05).

Within the CKD group over a six-month observation period, the serum concentrations of TMAO, creatinine, and SDMA were significantly higher in non-survivors than in survivors (Mann–Whitney U test; *p* < 0.05: 0.0142, *p* < 0.01, *p* < 0.001; Figure 8A–C). The median serum TMAO concentration was 137.0 ng/mL (IQR: 87.26–165.2) in deceased dogs and 70.18 ng/mL (IQR: 27.86–84.50) in surviving dogs. In contrast, serum cystatin C levels did not differ significantly between survivors and non-survivors within the CKD group (Mann–Whitney U test; *p* > 0.05; Figure 8D).

**Figure 9:**
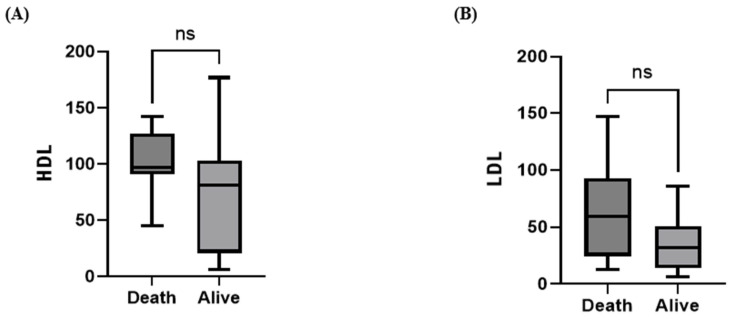
Box plot comparing the median HDL (**A**) and LDL (**B**) concentrations between the non-survivor group (*n* = 13) and survivor group (*n* = 17) at 6 months in the CKD group. The serum HDL and LDL concentrations in non-survivors were not statistically significantly higher than in survivors within the CKD group (Mann–Whitney U test; *p* > 0.05).

When comparing the survivor and non-survivor groups, the median values of both HDL and LDL showed an increasing trend in the non-survivor group; however, no statistically significant differences were observed (Figure 9).

### 3.9. Comparison of Prognostic Accuracy Through ROC Curve Analysis

**Figure 10:**
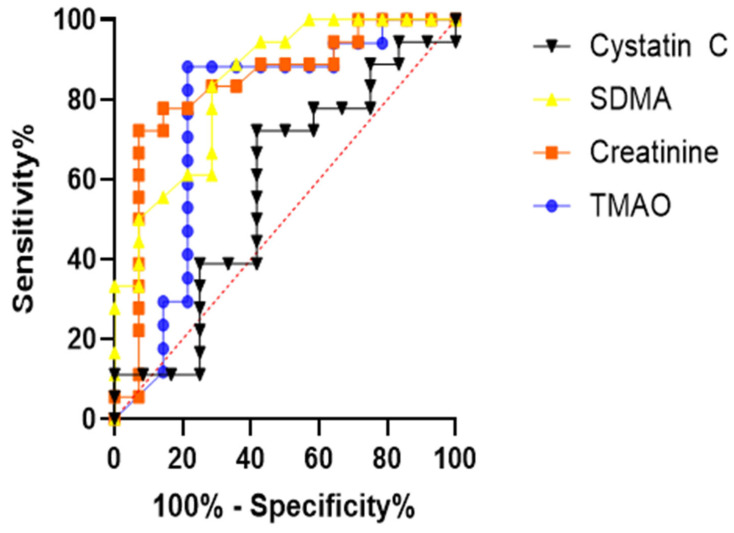
ROC curves of TMAO, creatinine, SDMA, and cystatin C concentrations to confirm their prognostic accuracy for mortality in dogs with CKD. ROC curve for predicting mortality in dogs with CKD; TMAO (AUC = 0.7563), creatinine (AUC = 0.8333), SDMA (AUC = 0.8393), and cystatin C (AUC = 0.5741) compared with survivor dogs.

An ROC curve analysis performed to predict mortality in the CKD group demonstrated significant predictive ability for TMAO (AUC = 0.7563; * *p* < 0.05; 0.0142), creatinine (AUC = 0.8333; ** *p* < 0.01), and SDMA (AUC = 0.8393; *** *p* < 0.001), when comparing survivors and non-survivors. However, cystatin C (AUC = 0.5741; *p* > 0.05) did not show statistical significance in predicting mortality (Figure 10).

## 4. Discussion

TMAO has been reported to be associated with cardiovascular diseases (e.g., myocardial infarction, stroke, and atherosclerosis) in humans. Subsequent studies have demonstrated a proportional relationship between elevated TMAO levels and an increased risk and poor prognosis of cardiovascular disease. Notably, even after adjusting for traditional risk factors such as diabetes mellitus and hypertension, elevated TMAO concentrations were associated with an increased risk of cardiovascular events [15].

In patients with CKD, TMAO concentrations significantly increase with advancing stages, demonstrate a negative correlation with GFR, and are associated with increased mortality rates [16,17]. Moreover, in individuals with normal kidney function, elevated TMAO levels are associated with up to a threefold increased risk of developing CKD [18].

In the present study, the serum creatinine, SDMA, and TMAO levels showed statistically significant differences, consistent with previous findings in human studies. Although cystatin C also exhibited statistically significant differences, these differences were not as strongly significant as those observed for TMAO, creatinine, and SDMA. Consistent with the findings of prior studies, cystatin C demonstrated lower reliability for detecting a reduced GFR than other biomarkers, possibly due to variability influenced by age, breed, and body fat percentage, as well as the lack of standardized testing methods in veterinary medicine [6]. These results suggest that TMAO may be superior to cystatin C for diagnosing CKD in dogs.

Comparisons of TMAO levels between the controls and dogs with different stages of CKD revealed statistically significant differences between the control group and each CKD stage group. Although the median TMAO values tended to increase with advancing CKD stage, these differences were not statistically significant among stages, likely due to the limited sample size.

For HDL and LDL, only LDL was significantly elevated in the CKD group compared with the control group, consistent with previous human studies reporting increased LDL levels in nephrotic syndrome due to reduced clearance and increased synthesis [19]. In addition, Pearson correlation analysis revealed a weak positive correlation between HDL and TMAO, whereas LDL showed an increasing trend with increasing TMAO concentrations, but this was not statistically significant. Previous studies have reported inconsistent correlations between TMAO and both HDL and LDL. Mechanistically, a negative correlation between HDL and TMAO can be explained by the role of HDL in cardiovascular disease prevention and the existence of a non-biliary cholesterol excretion route through the intestines, known as reverse cholesterol transport, which directly moves plasma cholesterol into the intestinal lumen independently of the primary hepatic–biliary pathway. In contrast, a positive correlation between HDL and TMAO may be explained by increased microbial diversity in the gut microbiota [20]. Furthermore, previous research on patients with CKD has demonstrated both high and low HDL levels, indicating the need for large-scale studies to further elucidate the relationships among CKD, HDL, LDL, and TMAO [21].

Within the CKD group, comparisons based on diet, age, antibiotic usage, and GI symptoms revealed no statistically significant differences in TMAO levels. This finding aligns with prior human CKD studies indicating that TMAO elevation occurs independently of age, diet, and other factors [22].

In this study, considering the hepatic metabolism of TMAO, we evaluated its correlation with ALT, ALP, GGT, and TBIL. For ALT, previous studies have shown inconsistent results, with some reporting a positive correlation due to simultaneous elevations of ALT and TMAO in fatty liver disease, while others indicated a negative correlation. Similarly, previous studies on ALP have been inconsistent, reporting either a positive correlation, no correlation at all, or even decreased ALP levels after TMAO administration, suggesting a hepatoprotective effect [23,24,25,26,27]. In the case of GGT, a previous study reported its tendency to increase alongside TMAO; however, this relationship was not statistically significant. With regard to TBIL, a previous study noted a decreasing trend with increasing TMAO, but this also was not statistically significant [24,26,27]. In this study, no significant correlations were identified between TMAO and ALT, ALP, GGT, or TBIL. Therefore, in canine CKD, TMAO may increase independently of hepatobiliary damage or function, specifically due to renal injury. Considering the known hepatic metabolism of TMA to TMAO, further large-scale studies investigating the relationships among hepatic enzymes, liver function, and TMAO are warranted. Additionally, it is important to identify factors that may affect TMAO production under various pathological conditions.

In human patients with CKD, individuals in the highest quartile of TMAO levels demonstrated a 2.8-fold increased risk of mortality over a 5-year follow-up period compared with those in the lowest quartile [28]. This elevated mortality risk was attributed to the induction of inflammation, fibrosis, and functional deterioration. Another meta-analysis similarly reported that higher circulating TMAO concentrations in patients with CKD were correlated with increased overall mortality, and this association remained significant even after adjusting for multiple confounding variables such as diabetes mellitus, blood pressure, blood lipid levels, renal function, and inflammatory markers [29].

Therefore, we aimed to evaluate whether these associations could be confirmed in dogs with CKD as prognostic markers. Consistent with human findings, our study demonstrated significantly higher serum TMAO levels in dogs in the non-survivor group than in the survivor group, thereby supporting the potential utility of TMAO as a prognostic biomarker in canine CKD.

In human medicine, TMAO is actively being investigated not only as a biomarker for renal function, in vitro kidney injury, and diagnostic and prognostic evaluation, but also as a therapeutic target.

At the cellular level, studies using human renal fibroblasts have demonstrated that TMAO promotes cell proliferation, collagen synthesis, and fibrosis induction [30]. Accordingly, extensive research has been conducted in human medicine to evaluate the reduction in TMAO through probiotics and dietary modifications [31,32]. One study demonstrated that reductions in TMAO levels resulted in decreased KIM-1, cystatin C, tubulointerstitial fibrosis, and renal collagen deposition and improved creatinine clearance [33].

Furthermore, one study in dogs with early-stage renal disease demonstrated a significant reduction in TMAO through dietary supplementation with betaine, which competitively inhibits choline (TMAO precursor), and soluble dietary fibers that promote beneficial gut microbiota growth [14].

Therefore, TMAO may serve not only as a biomarker but also as a potential therapeutic target and monitoring indicator. Given these promising aspects, further studies are warranted to clarify the therapeutic benefits associated with the reduction in TMAO levels.

Due to the inherent limitations associated with the retrospective nature of this study, GFR could not be measured in all cases. Second, the comparison of TMAO concentrations before and after treatment was not possible in all cases. Finally, as a pilot study with a limited sample size, the statistical significance of the findings was not highly robust. Therefore, further prospective studies with larger sample sizes are warranted to validate the benefits of measuring TMAO in canine CKD.

## 5. Conclusions

In this study, serum TMAO was demonstrated to indirectly measure GFR comparably to creatinine and SDMA for the diagnosis of CKD. In addition, TMAO could serve as a supportive diagnostic biomarker, with further potential as a prognostic monitoring marker in CKD. In this study, the specificity of TMAO for diagnosing CKD was measured at 86.67%, which is lower than the specificity of the current gold standard biomarkers for CKD, namely creatinine (100%) and SDMA (96.55%). As previously reported in human medicine, TMAO levels may be elevated not only in renal diseases but also in cardiovascular and endocrine disorders. Furthermore, studies have shown that even after managing cardiovascular and endocrine conditions in patients with renal failure, TMAO levels remain independently elevated in association with renal disease. In veterinary clinical practice, research on TMAO in dogs remains extremely limited. Therefore, further large-scale studies are warranted to determine whether TMAO levels increase independently in renal disease in dogs, and whether elevations also occur in diseases other than renal disorders. One of the limitations of conventional renal biomarkers is that creatinine is influenced by muscle mass and typically increases only after substantial renal function has already been lost. SDMA has been reported in some studies to be affected by conditions such as diabetes mellitus, sepsis, and thyroid disease. Cystatin C lacks standardized testing methods and its levels may vary depending on age, breed, and body fat percentage. Additionally, extra-renal conditions such as inflammation, thyroid disorders, and neoplasia can also increase cystatin C concentrations. To overcome these limitations of existing biomarkers, we measured TMAO. According to the International Renal Interest Society (IRIS), the optimal approach to utilizing renal biomarkers involves measuring multiple biomarkers and integrating their individual characteristics to improve diagnostic accuracy, prognostic assessment, and treatment monitoring. The ideal renal biomarker, as proposed by IRIS, should meet the following criteria. According to the International Renal Interest Society (IRIS), an ideal renal biomarker should be detectable in urine or blood, offering high accessibility and cost-effectiveness. It should accurately predict renal injury with high sensitivity and specificity, and provide information regarding the underlying etiology and the anatomical location of the renal lesion. Additionally, the biomarker should reflect the severity of renal injury and indicate whether renal damage is ongoing or if recovery is occurring. Furthermore, it should be capable of predicting the likelihood of renal recovery. TMAO is detectable in blood, offers high sensitivity and specificity, reflects the severity of renal injury, and is known to have potential utility in monitoring renal recovery. In this study, we successfully measured TMAO levels in blood and demonstrated that it had higher sensitivity and specificity than cystatin C. Therefore, measuring TMAO alongside creatinine and SDMA may serve as a useful adjunct for the diagnosis, prognostic evaluation, and therapeutic monitoring of renal disease. Future large-scale studies exploring TMAO as a therapeutic target are warranted, and the clinical management of TMAO may offer practical benefits for clinicians.

## Figures and Tables

**Table 1:** Signalments of all dogs included in the study.

Variables	CKD Group N = 32	Control N = 30
Median age (range)	11.12 ± 4.62	5.93 ± 3.89
Sex (n)	IF(2), SF(13)/IM(5), NM(12)	SF(6)/IM(15), NM(9)
Pomeranian	8	2
Maltese	4	6
Jindo	4	0
Poodle	3	1
Yorkshire Terrier	2	1
Pekingese	2	0
Golden Retriever	2	0
Cocker Spaniel	2	0
Bull Terrier	1	0
Bichon Frise	1	2
Coton de Tulear	1	0
Mixed	2	0
Beagle	0	16
Chihuahua	0	1
Schnauzer	0	1

**Table 2:** Cut-off value, sensitivity, and specificity of creatinine, SDMA, TMAO and cystatin C concentrations to confirm their diagnostic accuracy in dogs with CKD.

	Creatinine (mg/dL)	SDMA (ug/dL)	TMAO (ng/mL)	Cystatin C (mg/L)
Cut-off	1.350	14.50	53.78	0.7950
Sensitivity (%)	84.38	100	71.88	36.67
Specificity (%)	100	96.55	86.67	80

## Data Availability

The original contributions presented in this study are included in the article. Further inquiries can be directed to the corresponding authors.

## Abbreviations

The following abbreviations are used in this manuscript:

AUC	Area under curve
ALT	Alanine aminotransferase
ALP	Alkaline phosphatase
BUN	Blood urea nitrogen
CKD	Chronic kidney disease
FMO3	Flavin-containing monooxygenase 3
GFR	Glomerular filtration rate
GGT	Gamma-glutamyl transferase
GI	Gastrointestinal
HDL	High-density lipoprotein
LDL	Low-density lipoprotein
OD	Optical density
ROC	Receiver operating characteristic
SDMA	Symmetric dimethylarginine
TBIL	Total bilirubin
TMA	Trimethylamine
TMAO	Trimethylamine N-oxide
